# Redo-Transcatheter Aortic Valve Replacement with an Intra-Annular, Self-Expanding Prosthesis Within a Balloon Expandable Prosthesis: A Case Series

**DOI:** 10.1016/j.shj.2024.100324

**Published:** 2024-06-21

**Authors:** Ethan C. Korngold, Brandon M. Jones

**Affiliations:** Providence Heart Institute, Portland, Oregon, USA

**Keywords:** BASILICA, Navitor, Patient-prosthesis mismatch, Redo-TAVR, Valve-in-valve TAVR

As the population of patients treated with transcatheter aortic valve replacement (TAVR) expands, strategies to treat failed TAVR bioprostheses will be of growing importance. Potential challenges in redo-TAVR include the risk of coronary obstruction, coronary access, and patient-prosthesis mismatch with a nested implant.^1^ Traditionally, the Evolut system (Medtronic, Minneapolis, MN) has been well suited to redo-TAVR in a failed Sapien balloon-expandable valve (Edwards Lifescience, Irvine, CA) due to superior hemodynamics of the supra-annular valve compared to placement of a nested balloon-expanding valve, but depending on aortic root anatomy, this may put the patient at significant risk for coronary obstruction. The Portico and Navitor TAVR systems (Abbott, Santa Clara, CA) employ an intra-annular, self-expanding, repositionable, and retrievable design that maintains low leaflet height with hemodynamics similar to supra-annular valve designs.^2^ Specifically, it is uniquely beneficial to have the ability to more fully evaluate coronary perfusion with a root angiogram and echocardiography when the valve is partially deployed. This is in contrast to balloon-expanding valves, which are not retrievable, and the Evolut system in which the new leaflets are positioned significantly higher in the frame and do not achieve full expansion until the valve is completely released. There is limited published experience with redo-TAVR. A recent systematic review identified 160 patients, of whom only one had a Portico or Navitor implanted within a failed Sapien TAVR.^3^ In this series, we report 4 such patients with failed Sapien 3 prostheses with concerns for coronary obstruction; these patients were successfully treated with TAVR-in-TAVR Portico or Navitor implants.

Patients were evaluated by the heart team at our institution. While it was acknowledged that the Portico and Navitor systems are not currently Food and Drug Administration-approved for valve-in-valve use, after shared decision-making, we elected to proceed due to the lack of suitable alternatives based on an evaluation of the aortic root anatomy. Selected computed tomography angiography and fluoroscopic images and a summary of data for each patient are shown in Figure 1.

**Figure 1:**
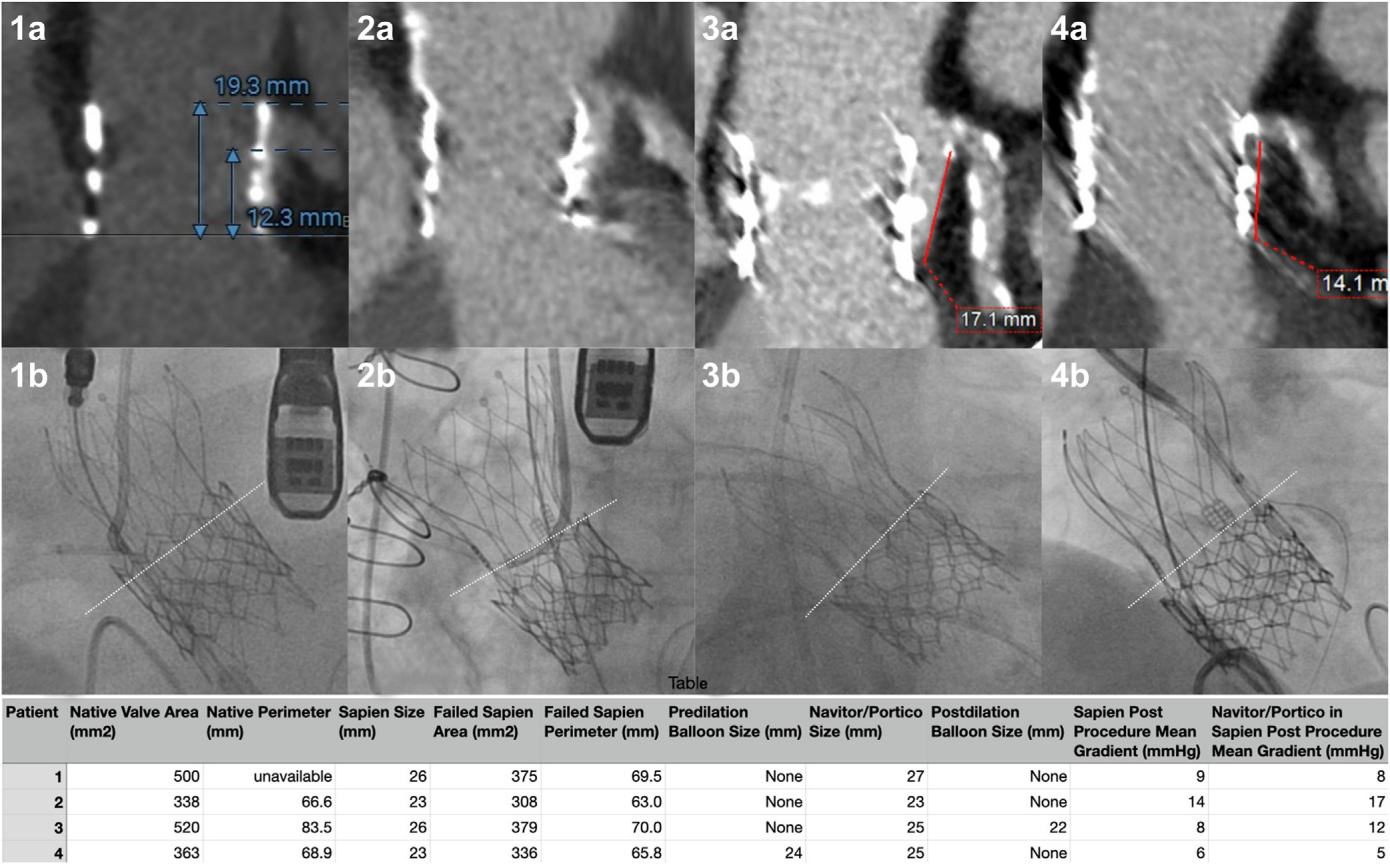
Selected preprocedural CTA images for 4 patients (1a-4a) demonstrating the relationship between the balloon-expandable TAVR frame, the sinotubular junction, and the left main coronary artery. Selected fluoroscopic images for 4 patients (1b-4b) showing the newly implanted self-expanding TAVR prosthesis within the balloon-expandable frame. The dashed line approximates the height of the newly implanted self-expanding valve leaflets. The table summarizes CTA measurements, balloon and valve sizes, and echocardiographic measurements for each patient. Abbreviations: CTA, computed tomography angiography; TAVR, transcatheter aortic valve replacement.

Patient 1 was an 84-year-old woman with severe aortic stenosis of a Sapien 3 26 mm valve 5.5 years after implant. The Bioprosthetic or Native Aortic Scallop Intentional Laceration to Prevent Iatrogenic Coronary Artery Obstruction (BASILICA) procedure of leaflet laceration was performed on the right and left leaflets prior to implantation of a 27-mm Portico valve approximately 6 mm below the initial prosthesis. By implanting the Portico valve low within the Sapien, it was expected that the upper edges of the new Portico leaflets (marked with a dashed line in Figure 1b) would fall below the BASILICA-created windows in the Sapien leaflets. This intentional low deployment would thus permit adequate coronary flow even if the Navitor were to deploy with the leaflet commissures directly in front of the BASILICA lacerations. No balloon valvuloplasty was performed. The postoperative mean echocardiographic gradient was 8 mmHg, similar to the initial Sapien postoperative mean gradient of 9 mmHg.

Patient 2 was a 79-year-old woman with severe aortic stenosis, 7.1 years after the implantation of a Sapien 3 23 mm valve. BASILICA was performed on the left coronary cusp, and the right coronary artery was protected with a guide and guide extender. Similar to the above case, it was hoped that the BASILICA combined with low implantation of the Portico would allow for left coronary perfusion without the need for coronary protection. A 23-mm Portico was implanted 1 mm below the prior valve. No balloon valvuloplasty was performed. The postoperative mean echo gradient was 17 mmHg, compared to the initial Sapien postoperative mean gradient of 14 mmHg.

Patient 3 was a 66-year-old man with multiple comorbidities and aortic stenosis, 5.5 years after implantation of a 26-mm Sapien 3. This was treated with a 25-mm Navitor valve implanted 2 mm below the initial implant. The Navitor was postdilated with a 22-mm balloon to ensure complete expansion. The postoperative mean echocardiographic gradient was 12 mmHg, compared to the initial Sapien postoperative mean gradient of 8 mmHg.

Patient 4 was a 78-year-old woman who developed mixed central and crescentic paravalvular regurgitation 1 year after implantation of a Sapien 3 23 mm valve. The Sapien was felt to be undersized with respect to the annulus. In this case, the strategy was to postdilate the Sapien valve with a 24-mm balloon so as to eliminate the paravalvular leak (recognizing that this would transiently worsen the central aortic regurgitation), followed by implanting a 25-mm Navitor, landing with an inflow 5 mm below the existing Sapien to minimize the risk of coronary obstruction. The left coronary artery was protected with a coronary guide and guide extender. The postoperative mean echocardiographic gradient was 5 mmHg, similar to the initial Sapien postoperative mean gradient of 6 mmHg. There was no residual aortic regurgitation.

All patients had successful redo-TAVR implantations without major complications and survived a 30-day follow-up. There were no instances of coronary obstruction or the need for snorkeled stents. BASILICA is useful to minimize the risk of coronary obstruction in redo-TAVR, but BASILICA alone may not be adequate to preserve coronary flow in the worst-case scenario of rotational misalignment where the new TAVR leaflet commissure blocks the BASILICA window.^4^ The intentional low implantation of the Navitor frame avoids this potential mechanism of coronary occlusion by positioning the Navitor leaflet and commissure material below the BASILICA windows. Despite the use of balloon valvuloplasty in only 2 of the 4 cases, the final mean gradient after redo-TAVR was similar to the initial balloon-expandable TAVR postoperative mean gradient, with no cases of patient-prosthesis mismatch. In our series, predilation was performed only to treat paravalvular regurgitation around the Sapien valve; otherwise, we do not think that predilation is an essential step for redo-TAVR with the Navitor system. Based on these cases, we think that using the computed tomography angiography measurements of area and perimeter of the failed Sapien is a reasonable approach to Navitor valve sizing, unless there is planned pre- or post-dilation larger than the original size of the Sapien valve, in which case a Navitor larger than the Sapien should be chosen. In our experience, the currently available Navitor system seems to be well-suited for redo-TAVR within a failed balloon-expandable valve, offering a repositionable, retrievable implant that can be comprehensively assessed by angiography and echocardiography before final release, an intra-annular design to minimize the risk of coronary obstruction, and excellent hemodynamics.

## Ethics Statement

This study was approved by the Providence St. Joseph Health institutional review board, with waiver of informed consent.

## Funding

The authors have no funding to report.

## Disclosure Statement

E. C. Korngold and B. M. Jones have received honoraria from Abbott and Edwards Lifesciences. There is no funding or support related to the design, conduct, or preparation of this report.
